# Comprehensive Pan-Cancer Analysis of Connexin 43 as a Potential Biomarker and Therapeutic Target in Human Kidney Renal Clear Cell Carcinoma (KIRC)

**DOI:** 10.3390/medicina60050780

**Published:** 2024-05-08

**Authors:** Huzi Xu, Xiuru Wang, Fan Zhu, Shuiming Guo, Zheng Chao, Chujin Cao, Zhihui Lu, Han Zhu, Meng Wang, Fengming Zhu, Juan Yang, Rui Zeng, Ying Yao

**Affiliations:** 1 Division of Nephrology, Tongji Hospital, Tongji Medical College, Huazhong University of Science and Technology, 1095 Jiefang Avenue, Wuhan 430030, China; xuhuzi1993@163.com (H.X.); wxrdoctor@163.com (X.W.); gsm91@163.com (S.G.); ccj9501@163.com (C.C.); zhuhan19901007@126.com (H.Z.); nishuihan2003@163.com (M.W.); zhufm1988@163.com (F.Z.); amy19861203@126.com (J.Y.); 2 Wuhan Central Hospital, Tongji Medical College, Huazhong University of Science and Technology, Wuhan 430030, China; colinzhu1991@gmail.com; 3 Division of Urology, Tongji Hospital, Tongji Medical College, Huazhong University of Science and Technology, 1095 Jiefang Avenue, Wuhan 430030, China; drchaoz@163.com; 4 Division of Nursing, Tongji Hospital, Tongji Medical College, Huazhong University of Science and Technology, 1095 Jiefang Avenue, Wuhan 430030, China; luzhihuiedu@163.com; 5 Division of Nutrition, Tongji Hospital, Tongji Medical College, Huazhong University of Science and Technology, 1095 Jiefang Avenue, Wuhan 430030, China

**Keywords:** Connexin 43, pan-cancer analysis, kidney renal clear cell carcinoma, prognostic biomarker, diagnostic biomarker

## Abstract

*Background and Objectives*: Connexin 43 (Cx43) is involved in the transfer of small signaling molecules between neighboring cells, thereby exerting a major influence on the initiation and progression of tumorigenesis. However, there is a lack of systematic research on Cx43 expression and its predictive role in clinical diagnosis and prognosis in pan-cancer. *Materials and Methods*: Several biological databases were used to evaluate the expression levels of GJA1 (encoding Cx43) and its diagnostic and prognostic significance in pan-cancer. We targeted kidney renal clear cell carcinoma (KIRC) and investigated the relationship between GJA1 expression and different clinical features of KIRC patients. Then, we performed cell-based experiments to partially confirm our results and predicted several proteins that were functionally related to Cx43. *Results*: The expression of GJA1 has a high level of accuracy in predicting KIRC. High GJA1 expression was remarkably correlated with a favorable prognosis, and this expression was reduced in groups with poor clinical features in KIRC. Cell experiments confirmed the inhibitory effects of increased GJA1 expression on the migratory capacity of human renal cancer (RCC) cell lines, and protein–protein interaction (PPI) analysis predicted that CDH1 and CTNNB1 were closely related to Cx43. *Conclusions*: GJA1 could be a promising independent favorable prognostic factor for KIRC, and upregulation of GJA1 expression could inhibit the migratory capacity of renal cancer cells.

## 1. Introduction

Connexins (Cxs), or gap junction proteins, are transmembrane proteins that form gap junctions (GJs) and hemichannels between two neighboring cells to transfer and exchange small molecules up to 1200 Daltons [1,2,3]. Twenty-one members of the connexin family have already been recognized in humans [4], each playing a vital role in maintaining organizational balance, regulating cell growth and facilitating cell differentiation [5,6,7,8]. Connexin 43 (Cx43), encoded by the GJA1 gene, is a highly studied and abundant member of the connexin protein family. It derives its name from its molecular weight of 43 kDa [9,10]. Fragmented forms of Cx43 have been implicated in exerting a vital role in the regulation of gene transcription and cell growth [11,12,13]. In addition, Cx43 expression has been observed to be associated with both cancer promotion and suppression, exerting significant influences on carcinogenesis, cancer progression, and metastasis [14]. For example, previous studies have shown that Cx43 expression was significantly decreased in gastric cancer, melanoma cancer [15], pancreatic cancer [16,17], lung cancer [18,19], chronic B-cell leukemia [20], prostate cancer [21], breast cancer [22,23,24,25], and thyroid cancer [9]. In addition, higher levels of Cx43 expression promoted metastasis [26,27,28] in many cancers, such as gastric cancer, melanoma, some brain cancers (astrocytoma, glioma) [29,30], breast tumors [22,23,25,31,32], and prostate cancer bone metastasis [21]. Notably, a study of 120 patients with thyroid cancer demonstrated that low Cx43 expression correlated with metastasis [9]. In recent years, a growing number of studies have demonstrated the diagnostic and prognostic value of Cx43 in various tumors. For instance, patients with cancers with high levels of Cx43 expression had a worse prognosis, such as non-muscle invasive bladder cancer, oral squamous cell carcinoma, primary breast cancer [33,34], melanoma, esophageal squamous cell carcinoma [9], and glioma tumors [35], while elevated levels of Cx43 expression were related with significantly longer survival rates in some types of breast cancer [25], colorectal cancer [36], head and neck squamous cell carcinomas [37], hepatocellular carcinoma [38], pancreatic cancer [16], and prostate cancer [39].

With the understanding of the role of Cx43 in normal and cancerous tissues, a number of Cx43 blockers have been developed for further mechanistic studies. Cx43 blockers are connexin mimetic peptides designed to mimic the extracellular or intracellular loop sequences of connexins to exert their blocking effects, including GAP19, GAP26, GAP27, Peptide 5, Tonabersat (Xiflam), α terminal junction proteinα (αCT1), and Danegaptide (GAP134) [40]. For example, upregulation of Cx43 expression in melanoma promoted anti-tumor immune killing by improving gap junction intercellular communication (GJIC) between cells, whereas blockade of GJIC function by Gap26 partially reversed this synergistic effect [41]. Our previously published research has also shown that GAP26 can significantly reduce Cx43 activity in renal tubular epithelial cells (TECs). Conversely, transforming growth factor -β1 (TGF-β1) could significantly increase gene expression of Cx43, which in turn increased the transporter activity of Cx43 in TECs [42].

Based on previous studies, it is clear that the relationship between Cx43 expression, cancer type, cancer stage, diagnosis, and prognosis of cancer patients is not always straightforward. Therefore, conducting a comprehensive and systematic analysis of the role of Cx43 in different tumors may reveal new clinical values of Cx43 in specific tumors.

Our study utilized several biological databases to evaluate the expression levels of GJA1 in pan-cancer, to analyze the diagnostic and prognostic significance of GJA1 in these tumor types, and to conduct cell experiments to further validate our findings. The results suggested that high GJA1 expression was remarkably correlated with a favorable prognosis, and this expression was reduced in groups with poor clinical features in KIRC. Cell experiments confirmed the inhibitory effects of increased GJA1 expression on the migratory capacity of human renal cancer (RCC) cell lines. Our research may have implications for the clinical prognosis of KIRC and further mechanistic research in the future.

## 2. Materials and Methods

### 2.1. Analysis of Cx43 Expression in Pan-Cancer

RNA sequencing (RNA-seq) expression profile data in level 3 HTSeq-Fragments Per Kilobase Million (FPKM) format of 33 tumor types and normal tissue samples were retrieved from The Cancer Genome Atlas (TCGA, http://portal.gdc.cancer.gov/, accessed on 7 September 2023)), and The Protein Atlas (http://proteinatlas.org/, accessed on 7 September 2023) databases. A total of 7946 tumor cases and 725 normal tissues were also obtained from TCGA for data analysis [43]. RNA-seq data were transformed from FPKM to transcripts per million (TPM) and then log2 converted. Statistical analysis was performed using R v3.6.3, and the visualization of the data was performed using the ggplot2 package. We used the Wilcoxon signed rank test for the analysis of data between two groups, which was considered to have statistical significance if *p* < 0.05.

### 2.2. Diagnostic Value Analysis

We evaluated the diagnostic significance of GJA1 expression in different types of cancer using a receiver operating characteristic (ROC) curve, and statistical analysis was performed using the pROC package. The area under the curve (AUC) is considered high accuracy if it is above 0.9, moderate accuracy if it is between 0.7 and 0.9, and low accuracy if it is between 0.5 and 0.7.

### 2.3. Survival Prognosis Analysis

We used Kaplan–Meier plots reflecting overall survival (OS), disease-specific survival (DSS), and progression-free interval (PFI) to evaluate the relationship between GJA1 expression levels in various cancer types and patient survival. The survival package and survminer package were conducted for statistical analysis and visualization, respectively. Hypothesis testing was performed using Cox regression, and correlations were regarded as having statistical significance if *p* < 0.05.

### 2.4. Associations between GJA1 Expression and Different Clinical Features in KIRC

We obtained RNA-seq data and relevant clinical data from the TCGA database and converted them from FPKM to TPM form, followed by log2 conversion. Data were analyzed using the Wilcoxon rank sum test, which was considered as having statistical significance when *p* < 0.05.

### 2.5. Univariate and Multivariate Cox Regression Analyses in KIRC

The correlations between GJA1 expression and clinical features in KIRC patients were assessed using univariate and multivariate Cox regression analyses, which were also used to evaluate the prognostic values (OS, DSS, and PFI) of GJA1 expression in different clinical subsets of KIRC. Corresponding statistical analyses were performed using the survival package.

### 2.6. Cell Lines and Cell Culture

Human renal tubular epithelial cell line HK-2, human renal cell carcinoma (RCC) lines 786-O, Achn, and OSRC-2 were obtained from Procell Life Science &Technology (Wuhan, China) and authenticated by the short tandem repeat (STR) method. HK-2, Achn, and OSRC-2 cells were incubated in DMEM/F12 medium (Keygen biotech, Nanjing, China), and 786-O cells were incubated in McCoy’s 5A medium (P/S) (Keygen biotech, Nanjing, China), with 10% fetal bovine serum (FBS) (Celligent, Hamilton, New Zealand) and incubated in sterile culture flasks at 37 °C with 5% CO_2_.

### 2.7. The mRNA Expressions of GJA1

Total mRNA was prepared from HK-2, 786-O, Achn, and OSRC-2 cell lines using Trizol reagent (Invitrogen, Waltham, MA, USA), followed by reverse transcription of the first-strand cDNA using the Transcription System (Vazyme, Nanjing, China). Quantitative PCR was performed on the ABI Step-One using the SYBR master mix (Vazyme, Nanjing, China). The 2^−ΔΔCt^ approach was used to calculate relative expression levels of mRNA, which were then normalized to the GAPDH expression levels. The forward primer for GJA1 mRNA was CAATCTCTCATGTGCGCTTCT, and the reverse primer for GJA1 mRNA was GGCAACCTTGAGTTCTTCCTCT. The GAPDH mRNA forward primer was GGAGCGAGATCCCTCCAAAAT, and the GAPDH mRNA reverse primer was GGCTGTTGTCATACTTCTCATGG.

### 2.8. Wound Healing Assay

For the wound-healing migration experiment, 786-O cells were spread evenly in six-well plates until they reached 80–90% confluence. The artificial wound was created in a cell monolayer; then, the control groups were replaced with 1% FBS, and the experimental groups were cultured with 1% FBS plus 5 ng/mL TGF-β1 (PeproTech, Rocky Hill, NJ, USA) or 0.02 μg/μL GAP26 (HY-P1082, MedChemExpress, Monmouth Junction, NJ, USA) or 0.04 μg/μL GAP26, respectively. Photographs of wound closure were taken at 0 h and after 12 h of the artificial wound treatment. Scratch healing rate = (final track area − initial track area)/initial track area × 100%.

### 2.9. DEGs between High and Low GJA1 Expression Groups in KIRC

Differentially expressed genes (DEGs) between the low and high GJA1 expression groups in KIRC were investigated using the deseq2 software package with R v3.6.3, and the results were examined using volcano plots drawn with the ggplot2 package, with thresholds of |log2 fold change (FC)| > 1.0 and adjusted *p* < 0.05. Furthermore, we used the STRING (https://string-db.org/, accessed on 7 September 2023) to analyze the protein–protein interaction (PPI) network of DEGs, with the following key parameters: a minimum required interaction score of “medium confidence (0.400)” and active interaction sources (“experiments, text mining, databases”). Hub genes in Cytoscape (version 3.9.1) were then analyzed using CytoHubba’s MCC algorithm.

## 3. Results

### 3.1. GJA1 Expression between Normal and Tumor Tissues

**Figure 1:**
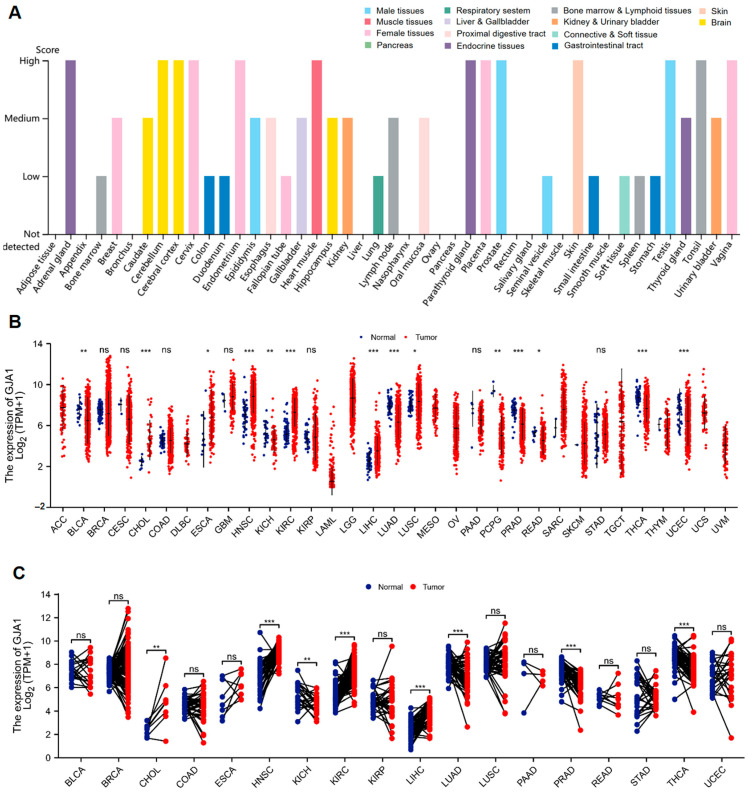
Cx43 expression in normal tissues and GJA1 expression in normal, tumor, and paracancerous tissues. (**A**) Cx43 expression in normal tissues with data from the Protein Atlas database. (**B**) Comparison of GJA1 expression between TCGA tumor tissues and normal tissues as controls based on data from TCGA. (**C**) Comparison of GJA1 expression between TCGA cancer samples and adjacent normal tissue samples as controls using data from TCGA. ns, *p* ≥ 0.05; * *p* < 0.05; ** *p* < 0.01; *** *p* < 0.001.

The Cx43 expression among normal tissues was detected using data from The Protein Atlas database. The following results revealed that the Cx43 expression was highly expressed in various normal tissues, including the adrenal gland, cerebellum, cerebral cortex, cervix, endometrium, heart muscle, kidney, parathyroid gland, placenta, prostate, skin, testis, tonsil, and vagina (Figure 1A). Then, we compared GJA1 expression levels between TCGA tumor samples and normal tissue samples (Figure 1B), and the results indicated that there was a significant upregulation of GJA1 expression in six tumor types, including bladder cholangiocarcinoma (CHOL), esophageal carcinoma (ESCA), head and neck squamous cell carcinoma (HNSC), kidney renal clear cell carcinoma (KIRC), liver hepatocellular carcinoma (LIHC), and lung squamous cell carcinoma (LUSC), while there was a significant downregulation in eight cancer types, including bladder urothelial carcinoma (BLCA), kidney chromophobe (KICH), lung adenocarcinoma (LUAD), pheochromocytoma and paraganglioma (PCPG), prostate adenocarcinoma (PRAD), rectum adenocarcinoma (READ), thyroid carcinoma (THCA), and uterine corpus endometrial carcinoma (UCEC). Furthermore, in TCGA tumors with paracancerous tissue as controls (Figure 1C), upregulation of GJA1 expression was found in four tumor types, including CHOL, HNSC, KIRC, and LICH, and downregulation of GJA1 expression was found in KICH, LUAD, PRAD, and THCA.

### 3.2. Diagnostic Significance of GJA1 in Cancer

**Figure 2:**
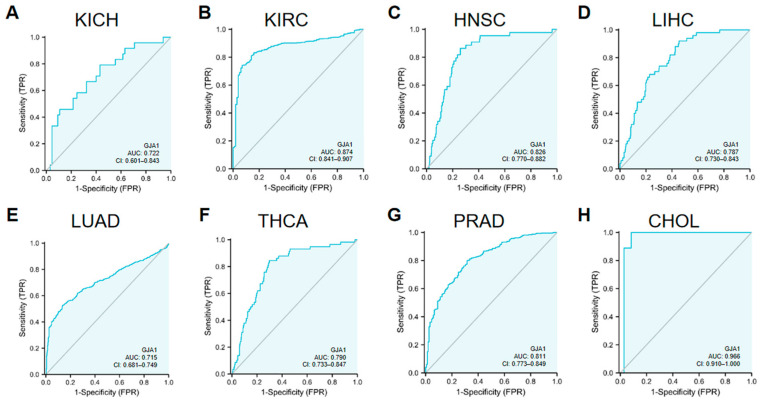
GJA1 expression in pan-cancer by ROC curve. (**A**) KICH; (**B**) KIRC; (**C**) HNSC; (**D**) LIHC; (**E**) LUAD; (**F**) THCA; (**G**) PRAD; (**H**) CHOL.

The diagnostic significance of GJA1 in various tumor types was evaluated by ROC curves (Figure 2) with findings revealing a certain accuracy (AUC > 0.7) of GJA1 when predicting eight different tumor types, including KICH (AUC = 0.722) (Figure 2A), KIRC (AUC = 0.874) (Figure 2B), HNSC (AUC = 0.826) (Figure 2C), LIHC (AUC = 0.787) (Figure 2D), LUAD (AUC = 0.715) (Figure 2E), THCA (AUC = 0.790) (Figure 2F), PRAD (AUC = 0.811) (Figure 2G), CHOL (AUC = 0.966) (Figure 2H). Among these evaluated cancer types, GJA1 predicted CHOL with high accuracy (AUC > 0.9).

### 3.3. Prognostic Significance of GJA1 in Cancer

**Figure 3:**
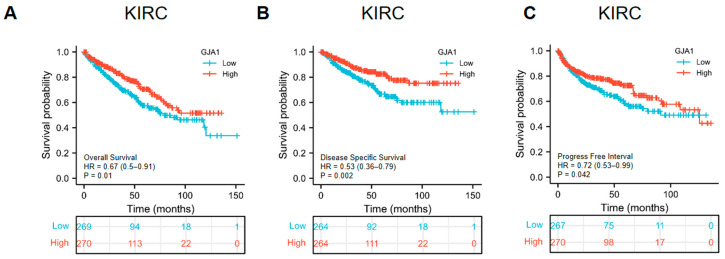
Prognostic significance of GJA1 in KIRC. (**A**) OS; (**B**) DSS; (**C**) PFI.

The associations between GJA1 expression and prognosis (OS, DSS, and PFI) in KICH, KIRC, HNSC, LIHC, LUAD, THCA, PRAD, and CHOL were investigated. The results indicated that higher GJA1 expression was related with improved prognosis in KIRC, as evident from the OS (hazard ratio (HR) = 0.67, *p* = 0.01) (Figure 3A), DSS (HR = 0.53, *p* = 0.002) (Figure 3B), and PFI (HR = 0.72, *p* = 0.042) (Figure 3C). For the other seven cancers, no DSS data were available for THCA. Additionally, the data for OS, DSS, and PFI in these seven tumors did not exhibit any statistically significant associations (Supplementary Figures S1–S3).

### 3.4. GJA1 Expression Is Associated with Various Clinical Features in KIRC

**Figure 4:**
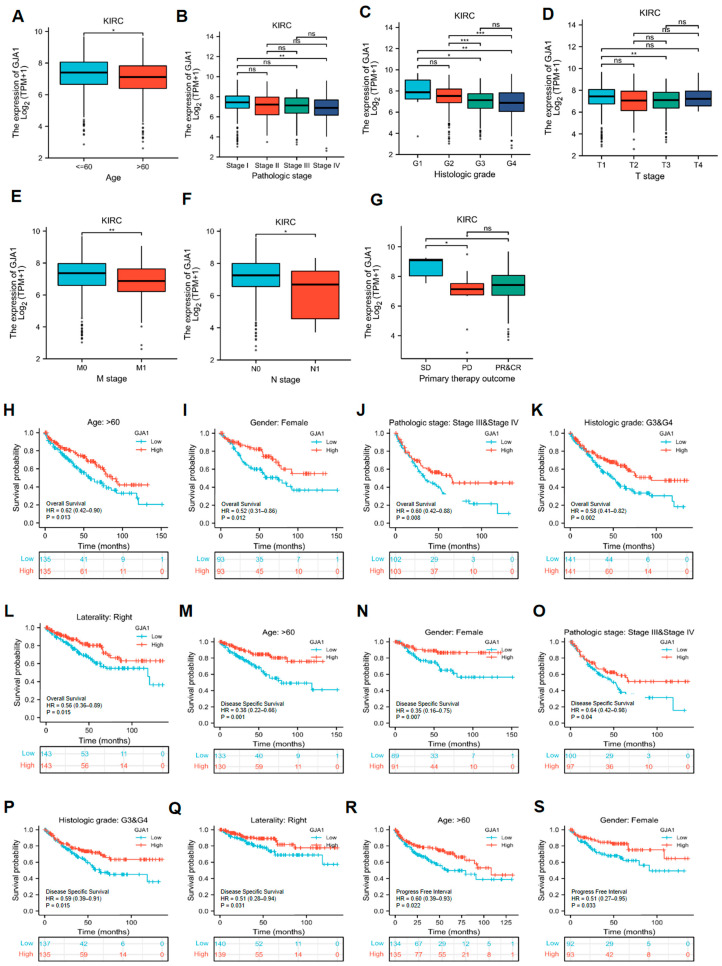
GJA1 expression and prognostic values in various clinical features in KIRC. (**A**) Age. (**B**) Pathologic stage. (**C**) Histologic grade. (**D**) T stage. (**E**) M stage. (**F**) N stage. (**G**) Primary therapy outcome. (**H**–**S**) Prognostic values in various clinical features in KIRC. (**H**,**I**) OS. (**M**–**Q**) DSS. (**R**,**S**) PFI. ns, *p* ≥ 0.05; * *p* < 0.05; ** *p* < 0.01; *** *p* < 0.001.

Our investigation included evaluating the connections between GJA1 expression and diverse clinical features in KIRC (Table 1). The results demonstrated that the relative expression of GJA1 was 7.3553 (6.6179, 8.002) in the age ≤ 60 group and 7.0655 (6.2779, 7.7754) in the age > 60 group with *p* value ≤ 0.05 (Figure 4A), and the expression of GJA1 decreased with increasing tumor pathologic and histologic stage (Figure 4B,C). For tumor TMN stage, the relative expression of GJA1 was 7.3921 (6.7015, 8.0274) in T1 group and 7.0226 (6.2713, 7.7501) in T3 group with *p* value ≤ 0.005 (Figure 4D); 7.3098 (6.584, 7.9256) in M0 group and 6.8012 (6.1927, 7.5609) in M1 stage group with *p* value ≤ 0.005 (Figure 4E); and 7.2388 (6.4801, 7.9459) in N0 group and 6.5712 (4.5042, 7.4705) in N1 group with *p* value ≤ 0.05 (Figure 4F). And the better the primary therapy outcome was, the higher the expression of GJA1 was (Figure 4G).

In addition, we also conducted a comparison of the relationships between the expression levels of GJA1 in KIRC and prognosis (OS, DSS, and PFI) in diverse clinical subsets The findings demonstrated that higher GJA1 expression was linked to improved OS in the following subgroups: age > 60 (HR = 0.62, *p* = 0.013) (Figure 4H), female gender (HR = 0.52, *p* = 0.012) (Figure 4I), pathologic stage: III and IV (HR = 0.60, *p* = 0.008) (Figure 4J), histologic grade: G3 and G4 (HR = 0.58, *p* = 0.002) (Figure 4K), and right laterality (HR = 0.56, *p* = 0.015) (Figure 4L). For DSS, higher GJA1 expression was related with a favorable prognosis in the subgroups of age > 60 years (HR = 0.38, *p* = 0.001) (Figure 4M), female gender (HR = 0.35, *p* = 0.007) (Figure 4N), pathologic stage: III and IV (HR = 0.64, *p* = 0.04) (Figure 4O), histologic grade: G3 and G4 (HR = 0.59, *p* = 0.015) (Figure 4P), and right laterality (HR = 0.51, *p* = 0.031) (Figure 4Q). Furthermore, for PFI, higher GJA1 expression was linked to a more favorable outcome in the subgroups of age > 60 years (HR = 0.60, *p* = 0.022) (Figure 4R) and female gender (HR = 0.51, *p* = 0.033) (Figure 4S).

### 3.5. Univariate and Multivariate Cox Regression Analyses in KIRC

Several clinical characteristics were included in univariate and multivariate Cox regression analyses, including age (≤60 vs. >60), gender (male vs. female), pathologic stage (Stage I and Stage II vs. Stage III and Stage IV), histologic grade (G1 and G2 vs. G3 and G4), T stage (T1 and T2 vs. T3 and T4), laterality (left vs. right), treatment history (no vs. yes), genetic background (no vs. yes), and GJA1 (low vs. high), to assess the association between GJA1 expression and clinical characteristics in KIRC patients. The results (Table 2) indicated that age (>60), pathologic stage III and stage IV, histologic grade: G3 and G4, laterality: left, genetic background (no), and low expression of GJA1 had significant correlations with the OS in KIRC as independent risk factors.

### 3.6. The Migratory Capacity of KIRC Cancer Cells Were Inhibited When the Expression of GJA1 Was Elevated

**Figure 5:**
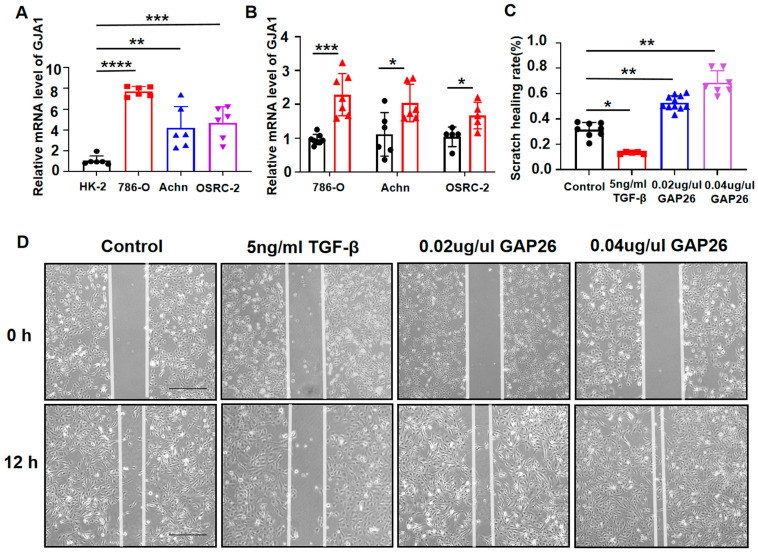
GJA1 expression was correlated with the metastatic ability in KIRC. (**A**) RT-qPCR. Levels of mRNA encoding GJA1 of HK-2, 786-O, Achn, and OSRC-2 by RT-qPCR. (**B**) RT-qPCR. Levels of mRNA encoding GJA1 of 786-O, Achn, and OSRC-2 when treated with TGF-β. (**C**,**D**) GJA1 suppressed migration of 786-O cell lines when treated with TGF-β and promoted 786-O cell lines migration when treated with GAP 26 as analyzed by scratch wound assay. Scale bar: 200 μm. Data are presented as the mean ± SEM, n = 6–9/group, ns, *p* ≥ 0.05; * *p* < 0.05; ** *p* < 0.01; *** *p* < 0.001; **** *p* < 0.0001.

We examined the expression of GJA1 in four cell lines, HK-2, 786-O, Achn, and OSRC-2. Compared to the HK-2 cell line, the expression level of GJA1 was increased 8-fold in the 786-O cell line and approximately 4-fold in the Achn and OSRC-2 cell lines, which was consistent with our TCGA database results (Figure 5A). Based on our previous studies highlighting TGF-β as an inducer of GJA1 [42], we performed RT-qPCR on the 786-O, Achn, and OSRC-2 cell lines and observed a significant rise in GJA1 expression in the TGF-β treated groups compared to the control, especially in the 786-O cell lines (Figure 5B). Furthermore, a scratch wound assay was employed to measure the effect of GJA1 on the migration of the 786-O cell lines. The result showed that higher levels of GJA1 significantly attenuated the metastatic ability of the 786-O cell lines as determined by the migration area, with a half reduction in cell migration rate (Figure 5C,D). To further evaluate the role of GJA1 in KIRC, we used specific Cx43 blockers, GAP26, after TGF-β. A scratch wound assay demonstrated that the GAP26-treated group had an almost 2-fold increase in the rate of scratch healing compared to the control group, particularly in the 0.04 μg/μL GAP26 group (Figure 5C,D). Therefore, it is plausible that high expression of GJA1 could potentially suppress the migratory ability of cancer cells in KIRC.

### 3.7. DEGs between GJA1 High Expression Group and Low Expression Groups in KIRC

**Figure 6:**
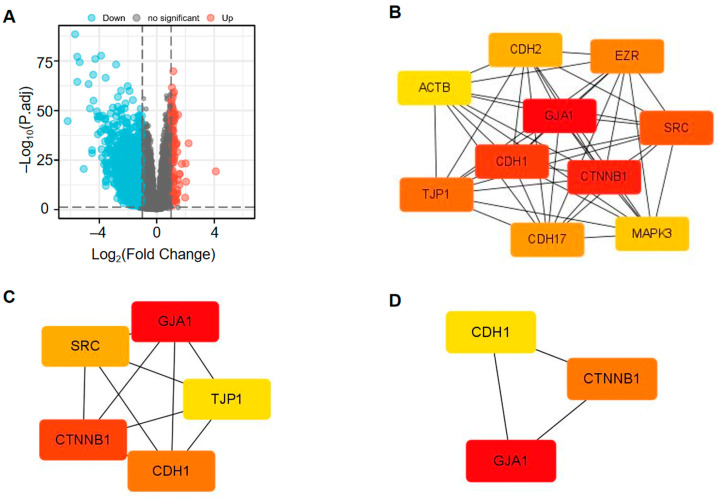
The volcano map and protein–protein interaction (PPI) network formation between the high and low expression groups of GJA1 in KIRC. (**A**) The volcano map of DEGs (red: upregulation; blue: downregulation; grey: no significance). (**B**–**D**) Hub genes of the PPI network with the MCC algorithm of CytoHubba in cytoscape.

In total, 1837 DEGs were obtained using the criteria of |log2 fold change (FC)| > 1.0 and adjusted *p*-value < 0.05, with 98 genes showing upregulation and 1739 genes showing downregulation (Figure 6A). In addition, the 10 most highly co-expressed genes of DEGs were identified (Figure 6B), namely, CDH1, CDH2, CDH17, EZR, SRC, MAPK3, TJP1, ACTB, GJA1, CTNNB1. Among them, CDH1, SRC, TJP1, GJA1, CTNNB1 were the top five hub genes (Figure 6C). Furthermore, CDH1, GJA1, CTNNB1 were the top three hub genes (Figure 6D).

## 4. Discussion

The results of our study highlighted a significant upregulation of GJA1 expression in six human tumor types: CHOL, ESCA, HNSC, KIRC, LIHC, and LUSC. Conversely, GJA1 expression was decreased in other eight tumor types, including BLCA, KICH, LUAD, PCPG, PRAD, READ, THCA, and UCEC. Additionally, when comparing the GJA1 expression in TCGA tumors with their corresponding paracancerous tissues as controls, significant upregulation was observed in four tumor types: CHOL, HNSC, KIRC, and LICH, while downregulation was observed in KICH, LUAD, PRAD, and THCA. These findings align with previous studies, highlighting the dual effects of Cx43 in diverse cancer types, where it can either act as a tumor-promoting gene or a tumor-inhibiting gene [37].

Recently, Cx43 has been characterized as a valuable diagnostic and prognostic biomarker in various cancers [14]. In our study, we employed ROC curve analysis and Kaplan–Meier survival curve analysis to assess the diagnostic and prognostic significance of GJA1 in pan-cancer. The results revealed that GJA1 was accurate (AUC > 0.7) in predicting eight types of cancer, especially highly accurate (AUC > 0.9) in predicting CHOL. Moreover, GJA1 showed a significant relationship with OS, DSS, and PFI in KIRC, indicating that patients with tumor expressing higher levels of GJA1 had a favorable prognosis. These findings indicate that GJA1 holds substantial diagnostic significance in KIHC, KIRC, HNSC, LIHC, LUAD, THCA, PRAD, and CHOL, and may be employed as a prognostic biomarker in KIRC.

Additionally, the associations between GJA1 expression levels in KIRC and several clinical characteristics were investigated: age, gender, pathologic stage, histologic grade, laterality, and primary therapy outcome. The results suggested that low GJA1 expression levels were related with unfavorable clinical features: age > 60 years, pathologic stage IV, histologic grade G3/4, presence of metastasis, and primary therapy outcome of PD. Conversely, high GJA1 expression levels were related with improved OS, DSS, and PFI in various clinical subsets of KIRC. Additionally, using Cox regression analyses, we confirmed that age, clinical stage, histologic grade, laterality (right), and GJA1 expression were independent risk factors for OS in KIRC.

Through in-vitro cell experiments, we found that the overexpression of GJA1 could potentially suppress the migration of 786-O cell lines. Additionally, wound healing assays implied that the suppression of GJA1 expression significantly upregulated the metastatic ability of 786-O cell lines. As a consequence, a high expression of GJA1 in human KIRC may predict a better prognosis. In addition, protein–protein interaction (PPI) analysis predicted that CDH1 and CTNNB1 were closely related to the function of Cx43.

Connexins, as a family of large pore channel-forming proteins, have critical functions in facilitating the transfer of small signaling molecules between adjacent cells [6,7,11,44]. Recent advances in connexin research have revealed their crucial contribution to cancer development and progression through modulation of cell proliferation [45], apoptosis [46], and cellular localization [11,14]. In KIRC, the most well-studied connexin is connexin 32 (Cx32), which is also a member of the same connexin family as Cx43 with a different molecular weight [47]. Cx32 is considered to be a tumor suppressor gene in KIRC, and studies have shown that Cx32 ameliorated the malignant phenotype of metastatic KIRC cells by inhibiting src-dependent signaling and activating c-Jun NH2-terminal kinase (JNK) signaling [47,48,49]. However, the role of Cx43 in KIRC has rarely been reported. Emerging evidence has confirmed that Cx43 can act as a tumor suppressor in gastric cancer, melanoma, pancreatic cancer, lung cancer, chronic B-cell leukemia, prostate cancer, breast cancer, and thyroid cancer [9,15,16,17,18,20,21,22,23]. However, it should be noted that Cx43 can also promote invasion and metastasis in triple-negative breast cancer (TNBC), non-small cell lung cancer (NSCLC), and astrocytoma [50,51,52]. Available studies have suggested that Cx43 plays a predominantly inhibitory role in KIRC, which is consistent with our study. For example, a study reported that all-trans retinoic acid (ATRA) could reverse the downregulation of Cx43 expression induced by exposure to 12-O-tetradecanoyl-phorbol-13-acetate (TPA) and dimethylnitrosamine (DMN), thereby preventing renal carcinogenesis [53], and vitamin D3 may also prevent KIRC by promoting GJIC during carcinogenesis [54]. However, these studies were only at the level of preclinical functional validation, and the expression of Cx43 in KIRC and its relationship with the prognosis of KIRC patients were still not clear. Therefore, our study clarified that Cx43 is highly expressed in KIRC, and high Cx43 expression is associated with better prognosis, and in-vitro experiments partially validated the ability of Cx43 to inhibit KIRC cell migration, which provides a direction for further exploration of the mechanism of Cx43 inhibition of KIRC, and whether there is any consistency or difference in the inhibitory effects of both Cx43 and Cx32 on KIRC also deserves in-depth investigation.

Generally, differential genes that are highly expressed in tumor tissues are regarded as oncogenes, but the interactions between tumors and the body’s immune microenvironment are complex, and immune cells, cancer-associated fibroblasts (CAFs) and endothelial cells (ECs) may communicate with cancer cells in the tumor microenvironment (TME) via functional GJIC; thus, highly expressed genes in some tumors are also associated with a better prognosis [55,56]. For example, it has been reported that the expression of chemokine ligand C-X-C motif chemokine ligand 11 (CXCL11) was upregulated in colon cancer tissue compared to normal tissue, and that tumor patients with high CXCL11 expression have a better prognosis, which may be associated with lower tumor-promoting immune cells and higher anti-tumor immune cells in the group with high CXCL11 expression [56]. In our own study, high expression of GJA1 in KIRC patients was associated with a better prognosis, which may be explained by the following points: (1) Cx43 is mainly localized to the cell membrane and mediates GJIC by forming gap junctions with neighboring cells, which are involved in cell migration, metabolism, and differentiation [32]. Under normal conditions, gap junction formation restricts cell migration to some extent, and increased expression of Cx43 may strengthen cellular junctions and restrict tumor cells from easily detaching from their original location, thereby decreasing the likelihood of cell migration [57]. This idea was partially supported by our subsequent cell experiments. (2) An important function of Cx43 is to mediate the transfer of small molecules between neighboring cells and also to transport anti-tumor drugs in vivo, which is closely linked to drug resistance following tumor chemotherapy [9,58]. Tumor cells with high Cx43 expression may be more likely to transport chemotherapeutic drugs into tumor cells and therefore be more sensitive to chemotherapeutic drugs and have better outcomes, but this hypothesis needs to be further tested in the future.

Analysis of the protein–protein interaction (PPI) network of DEGs between the GJA1 high expression group and the low expression group in KIRC revealed that CDH1 and CTNNB1 were closely related to GJA1. The E-cadherin (E-cad) gene (CDH1) is a calcium-dependent intercellular adhesion molecule that acts as a tumor suppressor, and its de-expression leads to a loss of intercellular adhesion accompanied by an increase in intercellular motility, which is strongly associated with the ability of tumors to infiltrate and metastasis. Dysregulation of its expression has also been found in some advanced cases of epithelial cancer [59,60]. β-catenin/CTNNB1, as an intracellular scaffolding protein, could interact with adhesion molecules (E-cadherin/CDH1, N-cadherin/CDH2, VE-cadherin/CDH5, and α-catenins), involved in cell adhesion, intracellular signaling, and transcription, and its mutations have been identified in lung, liver, prostate, gastric, and bladder tumors, involved in the malignant progression of various cancers [61,62]. Since all three molecules play an important role in tumor infiltration and migration by affecting the intercellular adhesion capacity, we speculate that high GJA1 expression may affect the intercellular adhesion of KIRC tumors and thus reduce the metastatic capacity of KIRC, either by affecting the expression of CDH1 and CTNNB1 or by interfering with the ability of the two interactions between E-cadherin and β-catenin, which needs to be verified by further in-vitro experiments.

There are still several limitations to our research that require acknowledgment. First of all, the evaluation of GJA1 expression across various cancers was based solely on data from the TCGA, The Protein Atlas, TISIDB, and STRING databases. Due to some data bias in database-derived studies, actual results may differ from the real world. For example, tumor purity is significantly correlated with the genomic differences and clinical characteristics of tumor patients, and the TCGA database suggests that 60% purity is sufficient to distinguish tumor components from non-tumor components; thus, tumor purity could introduce systematic bias into the results of tumor studies based on genomic analysis, and assessing tumor purity may reduce analytical bias [63,64]. Secondly, it is important to note that Cx43 may have diverse roles at different stages of tumor development, while our study focused primarily on its role at the transcriptional level, neglecting other crucial levels such as post-transcriptional regulation, protein synthesis, and post-translational modifications, and we only partially validated our findings at the gene level, lacking proteomic validation that may not fully reflect the functional impact of Cx43 in KIRC biology; thus, further research is required to fully understand its role in KIRC.

## 5. Conclusions

Our studies revealed that GJA1 holds diagnostic significance across various cancer types and can serve as a standalone favorable prognostic biomarker for KIRC, and increased GJA1 expression led to the inhibition of the migration capacity of renal cancer cells. These findings open up new avenues for future molecular research and highlight the prognostic significance of GJA1 in KIRC.

## Figures and Tables

**Table 1:** Clinical Features of KIRC patients.

Characteristic		Low Expression of GJA1	High Expression of GJA1	*p*
n		269	270	
T stage, n (%)				0.023 ^b^
	T1	121 (22.4%)	157 (29.1%)	
	T2	39 (7.2%)	32 (5.9%)	
	T3	103 (19.1%)	76 (14.1%)	
	T4	6 (1.1%)	5 (0.9%)	
N stage, n (%)				0.251 ^b^
	N0	122 (47.5%)	119 (46.3%)	
	N1	11 (4.3%)	5 (1.9%)	
M stage, n (%)				0.005 ^b^
	M0	202 (39.9%)	226 (44.7%)	
	M1	51 (10.1%)	27 (5.3%)	
Age, mean ± SD		61.62 ± 11.69	59.64 ± 12.43	0.058 ^a^

^a^: Student’s *t*-test, ^b^: Pearson’s chi-squared test. Data are presented as mean ± standard deviation (SD), median (25th–75th percentiles), or as a percentage.

**Table 2:** Univariate and multivariate Cox regression analyses of clinical features correlated with OS in KIRC.

Characteristics	Total (n)	Univariate Analysis		Multivariate Analysis	
		Hazard Ratio (95% CI)	*p* Value	Hazard Ratio (95% CI)	*p* Value
Age (≤60 vs. >60)	539	1.765 (1.298–2.398)	<0.001	1.590 (1.168–2.166)	0.003
Gender (Male vs. Female)	539	1.075 (0.788–1.465)	0.648		
Pathologic stage (Stage I and Stage II vs. Stage III and Stage IV)	536	3.946 (2.872–5.423)	<0.001	3.654 (2.656–5.025)	<0.001
Histologic grade (G1 and G2 vs. G3 and G4)	531	2.702 (1.918–3.807)	<0.001	1.731 (1.209–2.477)	0.003
T stage (T1 and T2 vs. T3 and T4)	539	3.228 (2.382–4.374)	<0.001	0.595 (0.331–1.070)	0.083
Laterality (left vs. right)	538	0.706 (0.523–0.952)	0.023	0.703 (0.519–0.951)	0.022
Treatment history (no vs. yes)	532	4.401 (3.226–6.002)	<0.001	1.223 (0.066–22.723)	0.893
Genetic background (no vs. yes)	533	0.156 (0.049–0.495)	0.002	0.094 (0.010–0.848)	0.035
GJA1 (low vs. high)	539	0.805 (0.719–0.901)	<0.001	0.758 (0.656–0.976)	0.030

## Data Availability

All data generated or analyzed during this study are included within the article.

## Supplementary Materials

The following supporting information can be downloaded at: https://www.mdpi.com/article/10.3390/medicina60050780/s1, Figure S1: Associations between GJA1 expression and the prognosis (OS) in cancer. (A) KICH; (B) HNSC; (C) LIHC; (D) LUAD; (E) THCA; (F) PRAD; (G) CHOL. Figure S2: Associations between GJA1 expression and the prognosis (DSS) in cancer. (A) KICH; (B) HNSC; (C) LIHC; (D) LUAD; (E) PRAD; (F) CHOL. Figure S3: Associations between GJA1 expression and the prognosis (PFI) in cancer. (A) KICH; (B) HNSC; (C) LIHC; (D) LUAD; (E) THCA; (F) PRAD; (G) CHOL.
