# Supplementary figures and images for: Comprehensive Pan-Cancer Analysis of Connexin 43 as a Potential Biomarker and Therapeutic Target in Human Kidney Renal Clear Cell Carcinoma (KIRC)
Source: Medicina (Kaunas). 2024 May 8;60(5):780. doi: 10.3390/medicina60050780 (PMC11123162; doi:10.3390/medicina60050780)

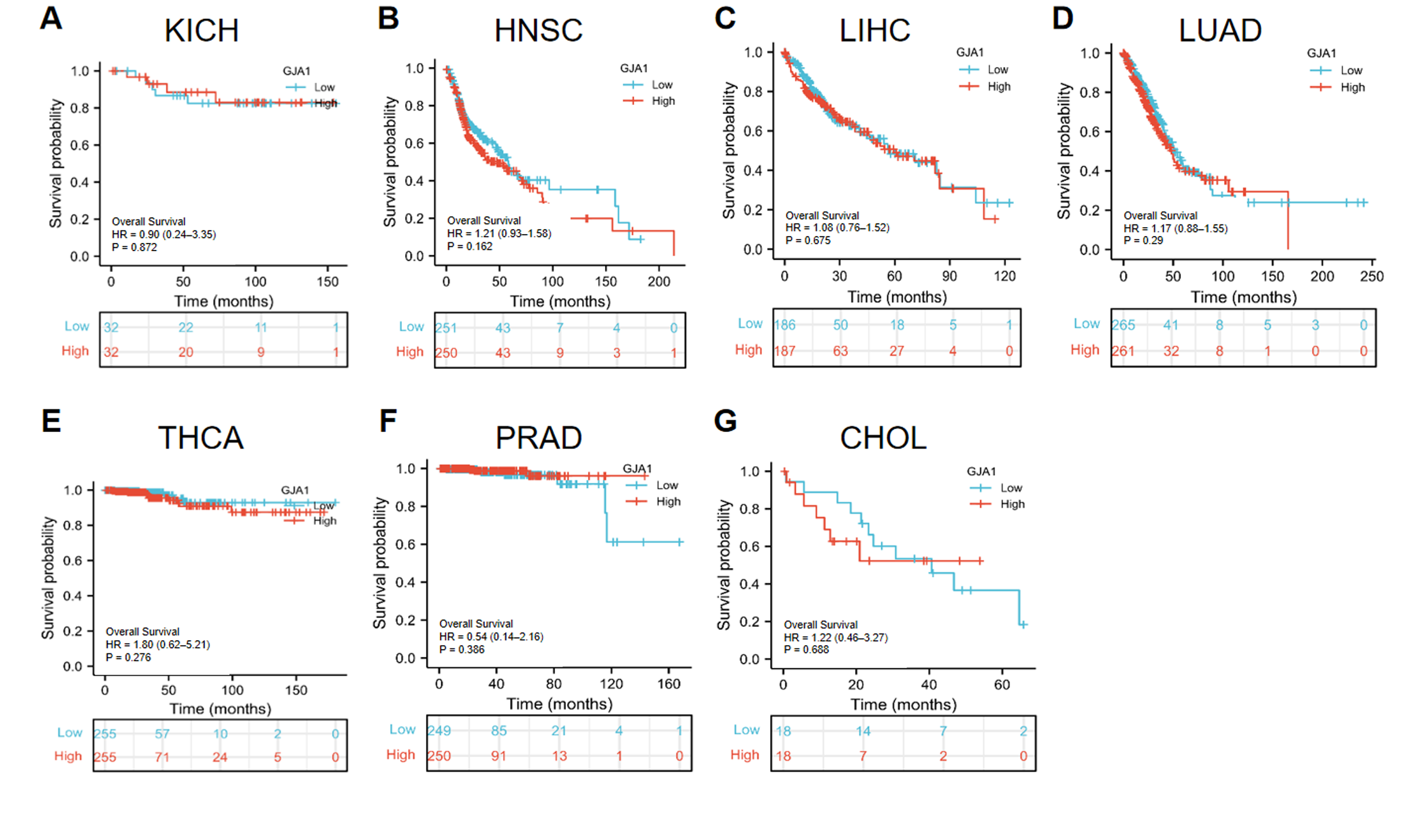

Supplement: Supplementary file 1 [file medicina-60-00780-s001.zip › Figure S1.tif]

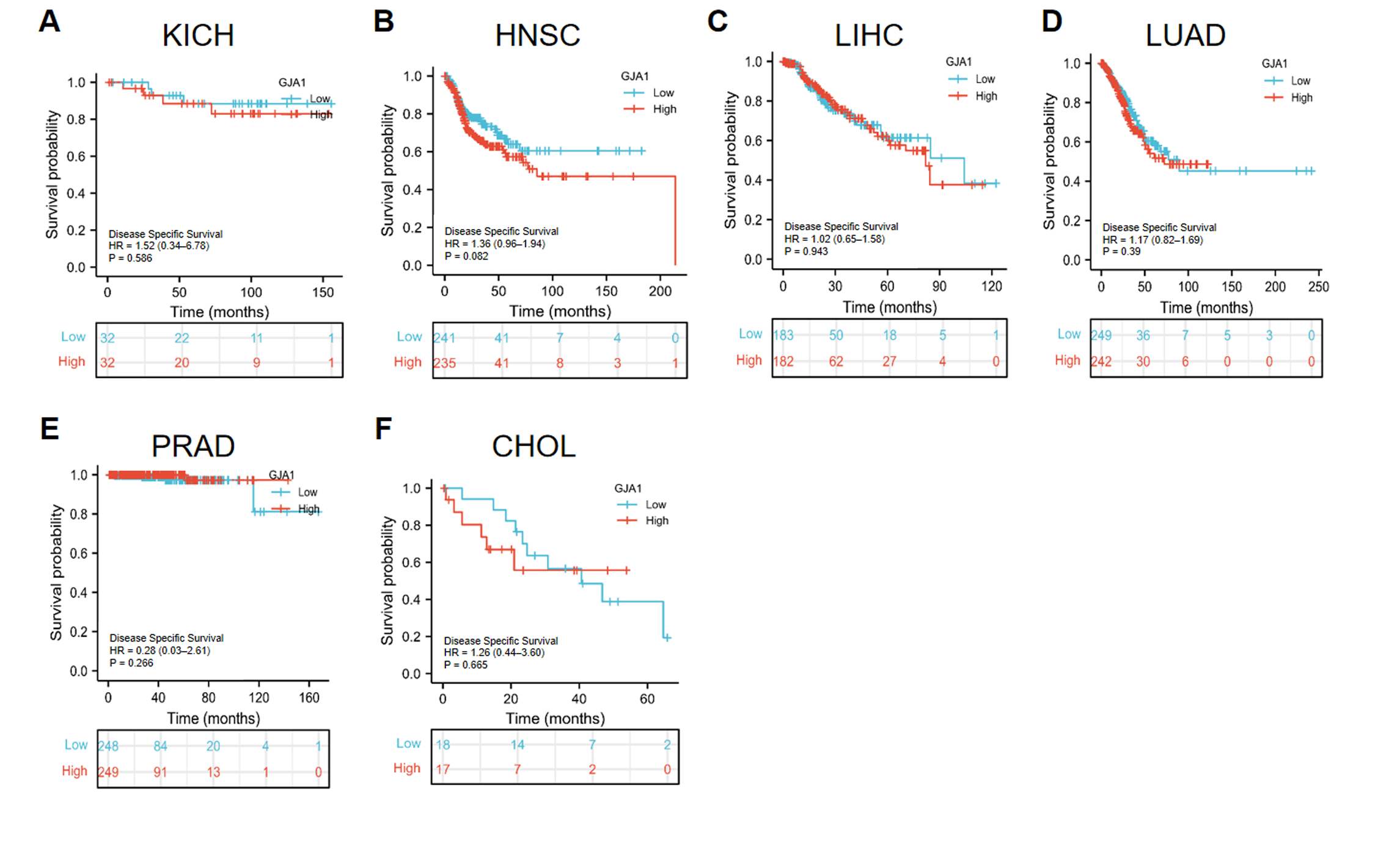

Supplement: Supplementary file 1 [file medicina-60-00780-s001.zip › Figure S2.tif]

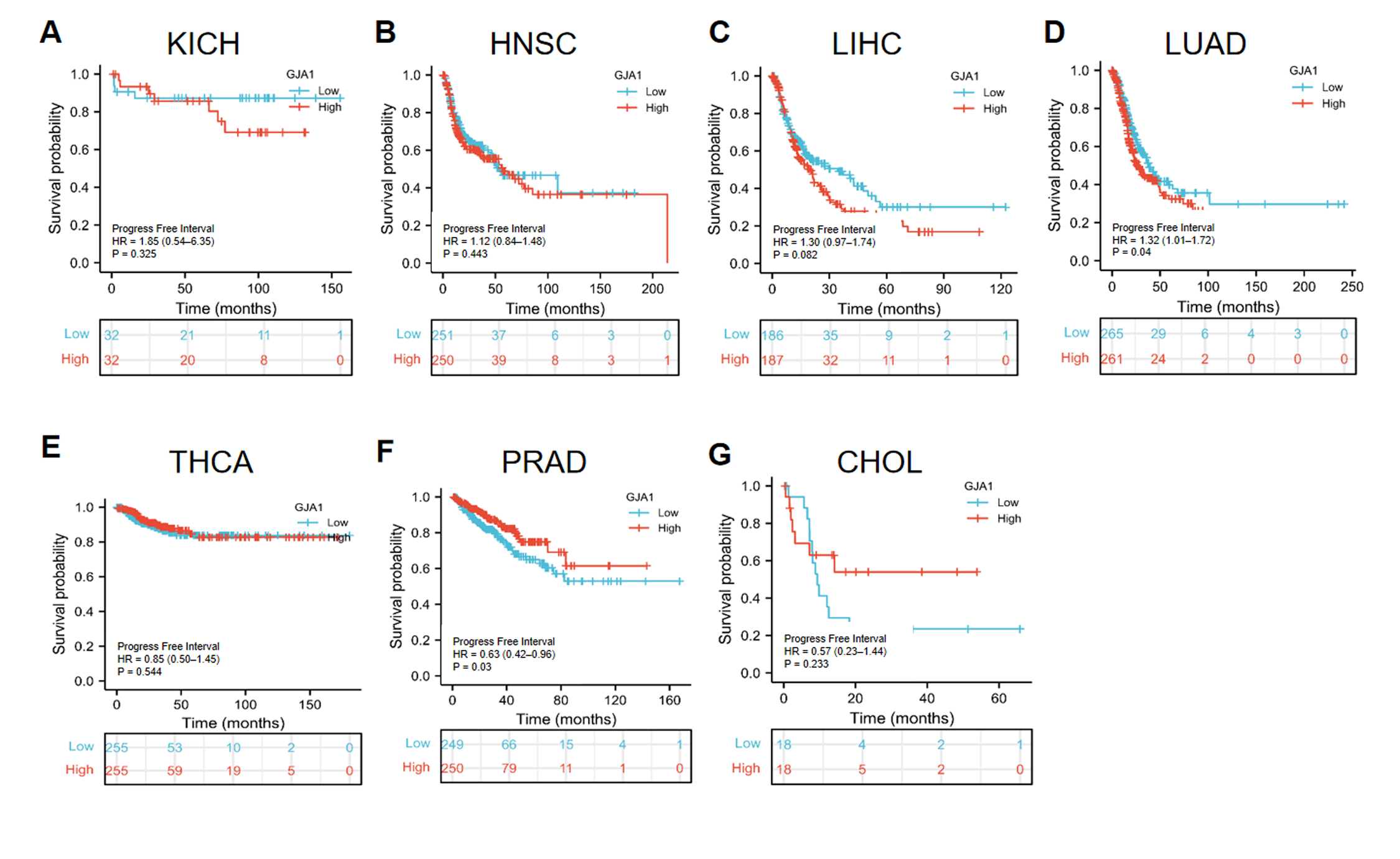

Supplement: Supplementary file 1 [file medicina-60-00780-s001.zip › Figure S3.tif]
